# Features of Self-Organization during the Process of Mindfulness-Based Stress Reduction: A Single Case Study

**DOI:** 10.3390/e25101403

**Published:** 2023-09-30

**Authors:** Günter Schiepek, Tatjana Marinell, Wolfgang Aichhorn, Helmut Schöller, Michael E. Harrer

**Affiliations:** 1 Institute of Synergetics and Psychotherapy Research, Paracelsus Medical University, 5020 Salzburg, Austria; 2 University Hospital of Psychiatry, Psychotherapy and Psychosomatics, Paracelsus Medical University, 5020 Salzburg, Austria; 3 Department of Psychology and Education Science, Ludwig-Maximilian University, D-80539 Munich, Germany; 4 Certified Mindfulness-Based Stress Reduction Teacher, 6020 Innsbruck, Austria; 5 Private Practice, 5020 Salzburg, Austria

**Keywords:** mindfulness, mindfulness-based stress reduction (MBSR), process monitoring, self-organization, dynamic systems, mechanisms of change, process feedback, outcome feedback

## Abstract

Compared to the extensive evidence of the effectiveness of mindfulness-based interventions, there is only a limited understanding of their mechanisms of change. The three aims of this study are (1) to identify features of self-organization during the process (e.g., pattern transitions), (2) to obtain an impression of the effects of continuous self-assessments and feedback sessions on mindfulness-related stress reduction, and (3) to test the feasibility of high-frequency process monitoring and process feedback. Concerning aim (1), the specific hypothesis is that change will occur as a cascade of discontinuous pattern transitions emerging spontaneously in the sense of not being a reaction to external input. This single case study describes changing patterns of multiple time series that were produced by app-based daily self-assessments during and after an 8-week mindfulness-based stress reduction program. After this MBSR program, the participant (a female nurse) continued the self-assessment and the mindfulness practice for a further 10 months. The results confirm findings on the positive effects of mindfulness programs for healthcare professionals, especially on coping with work-related stress. The analysis of the time series data supports the hypothesis of self-organization as a possible mechanism of change manifesting as a cascade of phase transitions in the dynamics of a biopsychosocial system. At the end of the year, the participant reported a beneficial impact of daily monitoring and systematic feedback on the change process. The results underline the feasibility and usefulness of continuous high-frequency monitoring during and after mindfulness interventions.

## 1. Introduction

Mindfulness and mindfulness-based interventions (MBIs) receive great interest from clinicians and researchers. There is a growing body of evidence on beneficial effects in both clinical and non-clinical populations, in settings like mental health care, education, different workplaces, and also in the military. MBIs reduce depression, anxiety, pain, and substance abuse [1,2]. MBIs can help to decrease stress [3], improve the coping abilities of health care professionals [4], support self-regulation [5] and self-care [6,7,8], and enhance well-being [9,10]. In medical disease management, MBIs are recommended to improve health-related quality of life [11].

Mindfulness-based stress reduction (MBSR) [12] and mindfulness-based cognitive therapy (MBCT) [13] are the two most widely available and evaluated mindfulness programs (MPs). MPs include an amalgam of mind-body practices and train different cognitive and affective skills that are known under the “umbrella term” of mindfulness [14].

Mindfulness’s very definition is multifaceted [15]. Bishop et al. [16] propose two components: One concerns the self-regulation of attention, allowing awareness of the experience in the present moment. The second component describes an attitude toward one’s own experiences that is characterized by curiosity, openness, and acceptance. In their IAA model (intention, attention, and attitude), Shapiro et al. [17] add intention as a third component. As a potential mechanism of mindfulness, they suggest that intentionally attending (A) with an open and non-judgmental attitude (A) should create a significant shift in perspectives, which is called “re-perceiving” or “decentering” [18,19,20]. In line with this conceptualization, we see mindfulness as a multidimensional construct, as represented in the factors of the *Comprehensive Inventory of Mindfulness Experiences* (CHIME, [21]). These dimensions include (1) awareness of inner and external experiences; (2) acting with awareness; (3) a decentered, accepting, non-avoiding, non-reacting, and non-judgemental attitude; (4) insightful understanding also concerning the relativity of thoughts; and (5) being conscious of one’s own intentions and remembering them as continuously as possible.

In this work, we are interested in the mechanisms contributing to the psychological effects of mindfulness practice. There are many hypotheses from a psychological or neurobiological perspective that we cannot report in detail here. The perspective we take in this study is on complexity science, which focuses not only on specific variables but on relations between variables. Thinking about relationships and networks contributing to state dynamics is deeply rooted in Buddhist philosophy and psychology. There are various similarities between the Buddhist worldview and thinking in complex systems [22]. Macy [23] compares Buddhism and general system theory [24]. To understand the emergent properties of a system—properties that do not exist at the level of the system’s parts but arise from the self-organization of the interconnected elements—it is necessary to look at the whole system embedded in its environment. Causality in open systems is not seen as a simple linear, unidirectional pathway; rather, it is characterized by dynamic interdependence involving mutual interactions and reciprocal effects, which in synergetics are modeled as mechanisms at a relative micro- and macro-level [25]. This is similar to Buddhist descriptions of “mutual causality”, “dependent co-arising”, and “dependent origination”. Nothing is independent, and the qualities of mental and physical processes seem to depend on certain conditions disappearing when the conditions disappear.

Empirical support for this view came from an experience-sampling study. In contrast to interpretations of mindfulness unidirectionally acting on affects in terms of a one-way relationship, the results of this study showed that the causal links between mindfulness and affect were bidirectional. Mindfulness influences affects, and vice versa [26].

Within Buddhist teachings, impermanence is one of the core messages about the nature of existence. It refers to the awareness and acceptance that all phenomena are transient and continuously changing. This concerns psychological phenomena like thoughts and emotions as well as states of mindfulness. In consequence, mindfulness should be conceptualized and measured as a process instead of the outcome given by point measures and stable traits [27].

More than 40 years ago, Kornfield [28] wrote: “*It seems important to emphasize that it is essential to recognize the nonlinear process of growth in meditation in order to construct proper research models. Unfortunately, much previous research has viewed meditation as if it would produce simple growth curves based on measuring one or more psychological or psycho-physiological variables over time*” (pp. 53–54). 

In formulating their “enactive approach” in cognitive science, Varela, Thompson, and Rosch [29] refer to the theory of “autopoetic” or self-organizing systems, relating the phenomenology of the living body to the Buddhist philosophical idea of “dependent origination”. The core assumption of enaction is that the living body is a self-organizing system [30]. Their concept of “embodied minds” conceptualizes mindfulness practices as skillful ways of enacting embodied states and behaviors in the world. This requires distinguishing between the causal enabling conditions, which include neural systems and cognitive processes that constitute mindfulness as a meaningful form of human experience [31]. Cognitive processes are constituted in the relational domain of the body coupled to its environment. 

Adopting the self-organization perspective of complexity science (e.g., synergetics and chaos theory), we have to respect the nonlinear interconnectedness (circular causality) of the involved variables and subsystems [25,32] and the dynamics of the system(s) under consideration. Self-organization is defined by spontaneous emergence (pattern formation) and change in patterns (pattern transition) in time (so-called phase transitions), which implies the empirical investigation of dynamics [25]. Self-organization is different from an input–output mechanism of change or a reaction to interventions, and a core assumption is a discontinuous change with occurring precursors like critical instabilities. Exploring change processes in mindfulness-based interventions as self-organized pattern transitions requires studying dynamics and patterns of behavior over time. Some studies started process research on dynamics. 

Baer et al. [3] assessed the change process of clients in an MBSR program once per week over seven weeks. Suelmann et al. [33] involved 29 experienced meditators in a study on present-moment experiences, with up to 5 assessments per day for 7 to 10 days. The study revealed that awareness of present-moment experiences was correlated with the intention to be mindful, with a positive mood at the moment, and not be busy, tired, or involved in social interactions. Andreotti et al. [34] used ecological momentary assessment (paper–pencil) on rumination, automatic pilot functioning, and attentional distractibility for 42 days, twice per day. The day-by-day measures of the participants in the intervention group (20 min daily formal mindfulness practice) and the control group were averaged. Over the resulting time series, a stepwise regression using a polynomial fit was calculated. Rumination, automatic pilot functioning, and attentional distractibility decreased more in the MBI group than in the control group, with a stable plateau in the middle of the MBI rumination trajectory. Aizik-Reebs et al. [35] applied network modeling to a short-time series of 15 experience sampling questions (5-point Likert scales), which were answered at the beginning and end of an MBI period (pre-post design, assessments twice per day, 3,5 days pre and post). The regularized partial correlation networks differed substantially from pre to post, with two nodes for each of the categories “equanimity”, “metacognitive processes”, “mood symptoms”, “negative self-referential processes”, and “emotion”. Also, the centrality indices of network nodes and node strengths were changed.

The complex systems perspective asks for a high-frequency investigation of dynamics, preferentially with equidistant measures, in order to identify the features of self-organization (e.g., discontinuous phase transitions, critical instability as a precursor of transitions, changes in complexity). Changes in complexity may not be a specific feature of mindfulness training or any psychotherapy approach but a general feature of self-organization (order parameter dynamics depending on control parameter shift). To achieve this and obtain sufficient lengths of time series data to investigate non-stationarity [36], the first steps of investigation have to consider *n* = 1 designs. Meanwhile, we have longitudinal research *and* big data (e.g., [37]), but if we intend to focus on specific research topics like mindfulness-based interventions, single case studies are still a design of preference. Another argument is not only the availability of data but also the importance of an idiographic approach to understand the specificity of a single case and to overcome the limitations of averaging data from many cases (group designs). Usually, trajectories of change are not only complex but also individual and distinct [36,38,39].

The study we are reporting in this article explores the process of change through daily self-assessments. The eight weeks of a MBSR program were the first period of a full year of data acquisition. This study was primarily conceptualized as a hypothesis-testing case study [40]. The *first aim* of the study was to test the hypothesis that the change process of a mindfulness-based intervention can be described as a self-organizing process characterized by sudden pattern transitions (phase transitions), which should emerge spontaneously in the sense of not being simply a reaction to external input [25,37]. In order to identify pattern transitions in time series, we applied a specific algorithm that not only respects shifting mean levels but also transitions of rhythms, complexity, or chaoticity (Pattern Transition Detection Algorithm, PTDA; [41]). Qualitative data from interviews with the participants should contribute to the *second aim* of the study, which is to answer the question of whether continuous self-assessment supports change processes and provides a better understanding of possible contributing factors. The compliance rate during a long period of ecological momentary assessment may reveal its feasibility in an eco-systemic context—the *third aim* of the study.

## 2. Materials and Methods

### 2.1. Participant

The subject of this study was a 40-year-old female health professional (nurse). She was a healthy person, not a patient, practicing different ways of meditation for one and a half years without any regular practice in mindfulness. She decided on the MBSR program in order to reduce her daily stress level and take some steps in her personality development. The mindfulness teacher was one of the authors (T.M.), certified as a mindfulness teacher for six years. From the beginning, the participant had positive expectations of mindfulness and personal development by accomplishing daily self-ratings using the *Synergetic Navigation System* (SNS).

Before the beginning of the MBSR program, the participant formulated three goals concerning her life: self-love, feelings of connectedness to her family, and trusting in life and God. Changes in these goals were targeted in the feedback sessions and occurred during one year of mindfulness practice.

### 2.2. Procedure

MBSR followed the standard curriculum by Kabat-Zinn [12,42] in a group setting of eight weekly sessions (2.5 h each) and one all-day retreat. The curriculum included mindful breathing, body scans, informal mindfulness, mindful yoga, and loving-kindness meditation, supplemented by exercises from the mindfulness-based cognitive therapy (MBCT) [13] protocol (three minutes of breathing space). An important ingredient of MBSR programs is repeatedly practicing formal and informal exercises at home and writing diaries in order to reflect on pleasant and unpleasant experiences and interpersonal events. These self-perceptions are explored in the group discussions. Another way of doing process feedback is part of MBSR through the dialogue and inquiry phases of the group sessions [43]. The group program proceeded during the first eight weeks of the monitoring period of one year. 

After the end of the MBSR program, she continued practicing 30–40 min every day (5 times per week in the first half of the year and 4–5 times per week in the second half) with elements of the program like mindful breathing, body scans, and loving-kindness meditation.

Using the time series diagrams of the change process, the progress and challenges of the participant were discussed together with the MBSR teacher (T.M.) and the initiator of the study (M.E.H.) at seven feedback sessions during one year. Based on these discussions, formal and informal practices were adapted. The interviews in the feedback sessions were semi-structured, guided by open questions on reaching goals and experiences concerning patterns of change. Important time series diagrams were discussed, analyzed, and interpreted together with her teacher. The average duration of the interview sessions was 2 h. There should be enough time for deepening insights and understanding the process.

The evaluation interview 7 months after the monitoring period was also realized as a semi-structured interview (the protocol of the semi-structured interview is available upon request from M.E.H.). It was on her retrospective experiences during the assessment period, especially on the daily assessments, the feasibility and effects of the integration of mindfulness practice in her daily life, and the effects of the feedback sessions. M.E.H. wrote a memory protocol after the evaluation interview (with some additions by T.M. and G.S.).

### 2.3. Measures

The process was monitored by a reduced version of the *Therapy Process Questionnaire* (TPQ, [44]), which included 20 out of 43 items. We excluded 23 items that were characteristic of inpatient or outpatient psychotherapy (e.g., alliance with professional therapists, relationship with fellow patients, symptom severity, and therapeutic progress). It should be noted that this MBSR study was conducted in a non-clinical context with a healthy person. The day-by-day intensity rating of each item was conducted by a visual analog scale, which was transformed to numeric values from 0 to 100 (e.g., from “not at all” to “very strong”).

The items were clustered into three categories corresponding to the factors of a former version of the TPQ: “Dysphoric Emotions”, with higher values corresponding to more worrying emotions and less positive emotions; “insight/new perspectives”; and “awareness/self-care” (see Table 1). The item “I felt tense and restless” as well as the item “I felt comfortable in my body” are indicators of body awareness and an important component of the mindfulness construct. Optimized self-regulation and coping with emotions should be the result of mindfulness practice [5]. The first four items of the awareness/self-care cluster (Table 1) are the items of the mindfulness/self-care factor of the current TPQ factor analysis [44]. All items, including those in the awareness/self-care cluster of the adapted TPQ, were approved by the participants in our study (dialogue-consensual validation). 

The factor values resulted from a three-step procedure: reverting the item values if necessary, averaging the item values of each measurement point, and applying a z-transformation to the resulting averaged time series. In addition to the quantitative assessments, a comment field was available for diary entries.

Changes in mindfulness were assessed pre and post-MBSR program and at the end of the assessment period by the *Comprehensive Inventory of Mindfulness Experiences* (CHIME) [21]. The eight factors of CHIME are “awareness of inner experiences”, “awareness of external experiences”, “acting with awareness”, “openness to experiences”, “acceptance and non-judgment attitude”, “decentering/non-reacting”, “insightful understanding”, and “relativity of thoughts”. 

Changes in self-compassion were assessed by the short form of the *Self-Compassion Scale* [45], German version [46]. Adequate psychometric properties of the scale are reported. Twelve items measure six components of self-compassion: positive aspects such as self-kindness, common humanity, and mindfulness, and negative aspects such as self-judgment, isolation, and over-identification. Items have to be rated on a five-point Likert scale ranging from 1 (almost never) to 5 (almost always). Coroiu et al. [47] proposed a two-factor model based on positive factors and negative factors.

**Figure 1:**
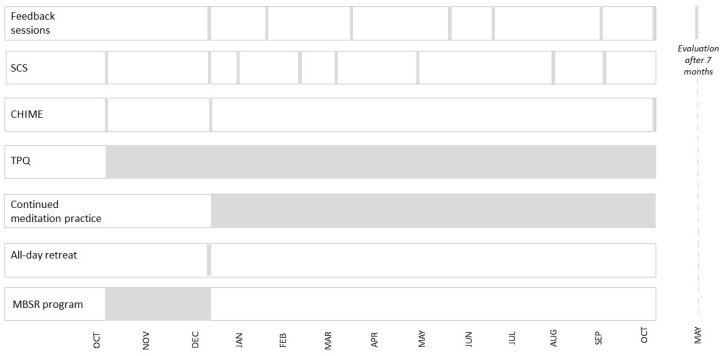
Time line illustrating the frequencies of feedback sessions and of administering outcome questionnaires (SCS: Self-Compassion Scale; CHIME: Comprehensive Inventory of Mindfulness Experiences) and of the process questionnaire (a modified and reduced version of the TPQ: Therapy Process Questionnaire). The TPQ was administered once per day, and the MBSR program and continued meditation practice were realized as mentioned above (2.2) during the respective period of time.

Figure 1 illustrates when and how frequently the process and outcome questionnaires were administered and when the feedback sessions were performed.

### 2.4. Data Analysis

The time series of the TPQ (items and factors) were analyzed by the **dynamic complexity (DC) algorithm** [25,48], which was developed to identify time-dependent degrees of complexity in short and coarse-grained real-world time series without further mathematical or parametric assumptions. DC multiplies a fluctuation measure and a distribution measure. Both can be calculated using discrete time series data with given data ranges and constant discrete time intervals between the dataset points (sampling frequency). The fluctuation measure (F) mirrors the amplitudes and frequencies of a time series, and the distribution measure (D) scans the scattering of values occurring within the range of possible values. In order to identify non-stationarity, DC is calculated in overlapping data windows moving over the time signal. In this study, we use a window width of 7 measurement points, which corresponds to one week. A sensitivity analysis testing other window widths than seven was realized [48] (a sensitivity analysis using different window widths is available upon request from the authors). It was shown that the DC measure is robust even if smaller window widths for calculation are used. The qualitative pattern of the complexity dynamics of a time series remains stable, independent of the window width. However, short windows produce doubtful results because the method uses turning points in a window (from increase to decrease or the other way around), which are lost in very short windows. Longer windows reduce the absolute complexity values, but the patterns of change remain. 

DC can be shown by a time series or color coding, as in *Complexity Resonance Diagrams*. Here, each line corresponds to an item of the process questionnaire. The maximum score of the dynamic complexity is depicted by a full red pixel, while all other values are graded according to that maximum (red = high, yellow = medium, and blue = low complexity). The x-axis represents the overlapping gliding windows.

**Recurrence plots (RP)** visualize and quantify recurrent, i.e., dynamically similar states within a time series [49,50]. Dynamic similarity is measured in terms of metric distances defined in the underlying state space. A single time series or even multiple time series are projected into a multidimensional state space by embedding procedures with a specific embedding dimension m and a time-delay parameter τ. The time-delayed embedding provides a reconstruction of phase-space profiles from a single one-dimensional time series or from a multidimensional time series. The dynamic similarity is represented in terms of metric distances d_ij_ = ‖ (${\vec{x}}_{i}$) − (${\vec{x}}_{j}$) ‖ defined in the reconstructed state space. Usually, recurrence plots are produced by binary matrices where an entry is 1 if d_i,j,_ ≤ ε; otherwise, it is 0 (where x_i_ and x_j_ are elements of the time series and ε is a threshold). This procedure depends on three parameters: the embedding dimension m, the time delay τ for taking values of the time series x_i,_ and the threshold ε, which defines the occurrence of recurrent (close) or distant (non-recurrent) state vectors in the m-dimensional embedding space. In our analysis, we selected a small embedding dimension of m = 3 and a small-time delay (τ = 1), which preserves the largest number of state vectors out of a given time series. Instead of applying the method of selecting a threshold ε, we used the Euclidian distance between all vector points $\vec{x}$ in the time delay embedding phase space. In consequence, the recurrence matrix R is equivalent to the distance matrix d = (d_i,j_). In the time x time recurrence matrix, the color of each pixel or cell reflects the dynamic similarity between all vector points, with dark blue representing identity or very short Euclidian distances between the vector points and red representing the longest Euclidian distance between any two vector points (rainbow color spectrum). 

**Dynamic Correlation Matrices:** Correlation matrices represent the inter-correlations of the time signals we are interested in (from one or different systems). In this case, we take the time series of the items in the process questionnaire. We represent the dynamics of the inter-correlations by taking into account the sequence of overlapping correlation matrices (running window; window width: 7 measurement points; overlap: 6, which corresponds to a step of 1). Changes are presented by a sequence of correlation matrices with color-coded correlations (from −1 [dark red] over 0 [white] to +1 [dark green]). A marker can be dragged along the time points to display the change in synchronization patterns over time.

The **Pattern Transition Detection Algorithm (PTDA):** The PTDA combines different methods of linear and nonlinear time series analysis in order to identify pattern transitions in a time series [41,51]. Change Point Analysis is applied to the original time series data (with reference to changing levels, variance, and linear trends) and to second-order measures as dynamic correlations and the lines of recurrence plots and **Time–Frequency Distributions (TFD)**. The method provides a probability estimate of a pattern transition (in physical terms, a phase transition) and visualizes where it occurs in the process.

As described [51], a valid pattern transition (PT) should be characterized by a clustering of change points (CPs) around the real pattern transition of the time series. This dispersion along the time series should be different from the dispersion of points that are placed randomly on the time series. Inspired by bootstrapping, random values were drawn from a discrete uniform distribution of the length of the respective time series with the *unidrnd* function implemented in Matlab. The number of random values drawn is equivalent to the number of change points found by the different methods for the respective time series. This is repeated 100 times. The spreading of the points of non-normal distributions can be assessed by the interquartile range (IQR). The IQR describes the number of points within the second and third quartiles of the data, i.e., the inner 50% around the median, and is a measure of the dispersion of the data comparable to the variance of normally distributed data. After calculating the IQR of the 100 sets of random CPs, the mean and the 95% confidence interval of these IQRs are calculated. If the IQR of the real data lies below the lower bound of the confidence interval of the equally distributed random data, the PT is considered significant. 

**Change Point Analysis (CPA)** [52] is applied to the raw data time series and the results of the analysis methods as dynamic complexity, line by line to recurrence plots and to Time–Frequency Distributions (second-order time series). The method is sensitive to changes in specific statistical properties of a time series. A time series x_i_ contains a change point if it can be split into two segments, x_1_ and x_2,_ such that C(x_1_) + C(x_2_) < C(x), where C represents the cost function. Here, C(x) = Nvar(x), where N is the number of time points of x_i_. In other words, a change point is detected between the segments x_1_ and x_2_ of a time series if the sum of the variance of the statistical property of interest, e.g., the mean of the segments, is smaller than the variance of this property of the whole time series; otherwise, no change point is detected. The function is able to detect changes in the mean, the variance, and the linear trend of a time series. In the PTDA, these three possibilities are applied by default to the original time series. For the application of the secondary methods, only changes with respect to the mean are assessed.

**Time–Frequency Distribution (TFD)** is a method to calculate and visualize the frequency of a signal (time series) as it changes with time [53]. In order to identify frequency changes, a moving window approach is implemented. Both time t and frequency ω are variables of a distribution P (t,ω), which describes the amplitude (energy) of the signal at each given t and ω. Here, we use the so-called Stockwell transform (S-transform), which is a combination of two common TFD methods: the short-time Fourier transform and the continuous wavelet transform [54]. It preserves the phase information available from the former method but uses the variable (i.e., not fixed) window length of the continuous wavelet method. For visualization, time and frequency are plotted on a plane (x: time, y: frequency), and color coding is used for the representation of the amplitude (energy) of the frequencies.

## 3. Results

**Figure 2:**
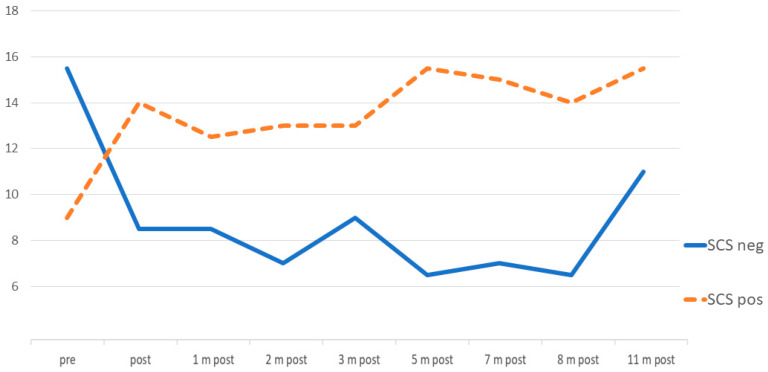
Self-Compassion at pre and post-intervention and seven follow-ups. SCS pos (orange dashed line): Self-kindness, common humanity, mindfulness. SCS neg (blue line): Self-judgment, isolation, over-identification. After the training period, the SCS was administered monthly.

Figure 2 shows the low-frequency assessment by the Self-Compassion Scale (SCS) during the process at pre and post-intervention and seven monthly follow-ups. Evidently, the biggest change occurs before and after the MBSR program: An increase in the positive factors (self-kindness, common humanity, mindfulness) and a decrease in the negative factors (self-judgment, isolation, over-identification).

The high-frequency change processes of our participants were assessed over 365 days, which is far more than the duration of the MBSR program at the first eight weeks of the assessment period. During this year, the participant filled in 262 daily self-ratings (71.8%) and wrote 55 diaries. 

**Figure 3:**
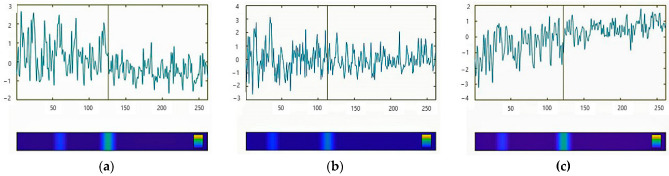
Time series of the factors (**a**) “Dysphoric Emotions”, (**b**) “insight/new perspectives”, and (**c**) “awareness/self-care”. X-axis: time in days; y-axis: z-transformed factor values. The vertical lines indicate the pattern transition of the process, as estimated by the Pattern Transition Detection Algorithm (PTDA).

Figure 3 illustrates the time series of all three *Therapy Process Questionnaire* (TPQ) factors (x-axis: time in days, y-axis: z-transformed factor values). The vertical lines indicate the pattern transition of the process, as estimated by the Pattern Transition Detection Algorithm (PTDA). The line corresponds to the bright turquois sector in the blue bar under the diagrams, indicating the highest probability of a pattern transition. The second, less important transition corresponds to the bright blue sector, which is a coincidence with the end of the MBSR group training.

**Figure 4:**
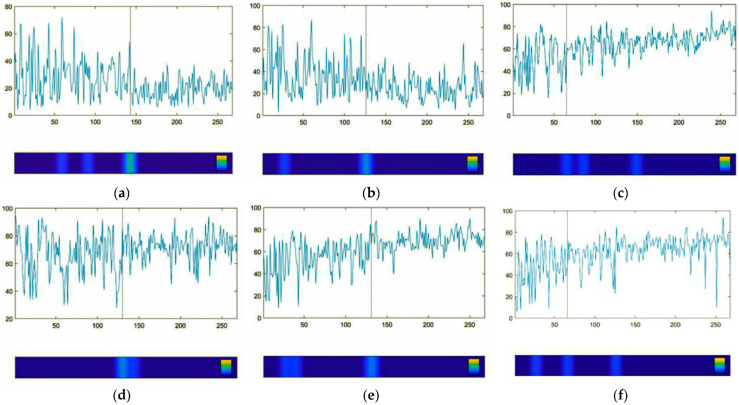
Time series of the TPQ items includes the following items: (**a**) “Today, I felt anxious”. (**b**) “Today, I felt tense and restless”. (**c**) “Today, I felt comfortable in my body”. (**d**) “Today, I experienced moments of happiness and lightheartedness”. (**e**) “Today, I treated myself with care and mindfully”. (**f**) “Today, I paid attention to my boundaries/limits”. X-axis: time in days; y-axis: item values (0–100). The vertical lines indicate the pattern transition of the process, as estimated by the Pattern Transition Detection Algorithm (PTDA). The line corresponds to the bright turquois sector in the blue bar under the diagrams, indicating the highest probability of a pattern transition. Lines in (**a**,**b**,**d** and **e**) correspond to the pattern transition as it was found in most of the time series (see also Figure 3). Lines in (**c**,**f**) correspond to a less frequently found pattern transition occurring at the end of the MBSR program.

Figure 4 shows a time series of six single items of the *Therapy Process Questionnaire* (TPQ) (x-axis: time in days; y-axis: item values 0–100). The six items are as follows: (a) “Today, I felt anxious”. (b) “Today, I felt tense and restless”. (c) “Today, I felt comfortable in my body”. (d) “Today, I experienced moments of happiness and lightheartedness”. (e) “Today, I treated myself with care and mindfully”. (f) “Today, I paid attention to my boundaries/limits”. The vertical lines indicate the pattern transition of the process, as estimated by the Pattern Transition Detection Algorithm (PTDA). The line corresponds to the bright turquois sector in the blue bar under the diagrams, indicating the highest probability of a pattern transition. Lines in (a), (b), (d), and (e) correspond to the pattern transition as it was found in most of the time series (see also Figure 2). Lines in (c) and (f) correspond to a less frequently found pattern transition occurring at the end of the MBSR program. We selected the items shown in Figure 4 for illustrative reasons. Upon request, the time series of the other items will be available, including the PTDA applied to the time series.

The time series mirrors the course of her mindfulness practice in a very detailed and differentiated way. In the dynamics of the factors and almost all items, it seems evident that she experiences progress in her well-being together with less worrying and more positive emotions, obtains more insight, develops perspectives on her life, and obtains access to her body and mindfulness. The Pattern Transition Detection Algorithm shows discontinuous jump-like transitions, depending on the item, at two times: In many items (the time series of the items) and in the time series of all three factors (see Figure 3 and Figure 4), the most important transition is at about day 135 (four months after she had finished the MBSR group training). A few items show the transition at the end of the group training. This corresponds to a two-step evolution. The results of the PTDA only indicate a transition if it is significant. This means that a significant transition was identified that respects not only mean level changes but also changes in complexity, rhythms, or even chaoticity at the same mean level of a time series, which is usually not respected by other methods.

This corresponds to the pre-post (after the MBSR program) follow-up differences in the factor scores of the *Comprehensive Inventory of Mindfulness Experiences* (CHIME): “awareness of inner experiences”: 20.0–21–22.0; “awareness of external experiences”: 16.5–17–22.0; “acting with awareness”: 11.0–17–19.0; “openness to experiences”: 12.5–17–15.0; “acceptance and non-judgment attitude”: 15.5–15–16.0; “decentering/non-reacting”: 17.5–23–26.0; “insightful understanding”: 17.5–17–19.0; and “relativity of thoughts”: 20.0–19–20.0. 

**Figure 5:**
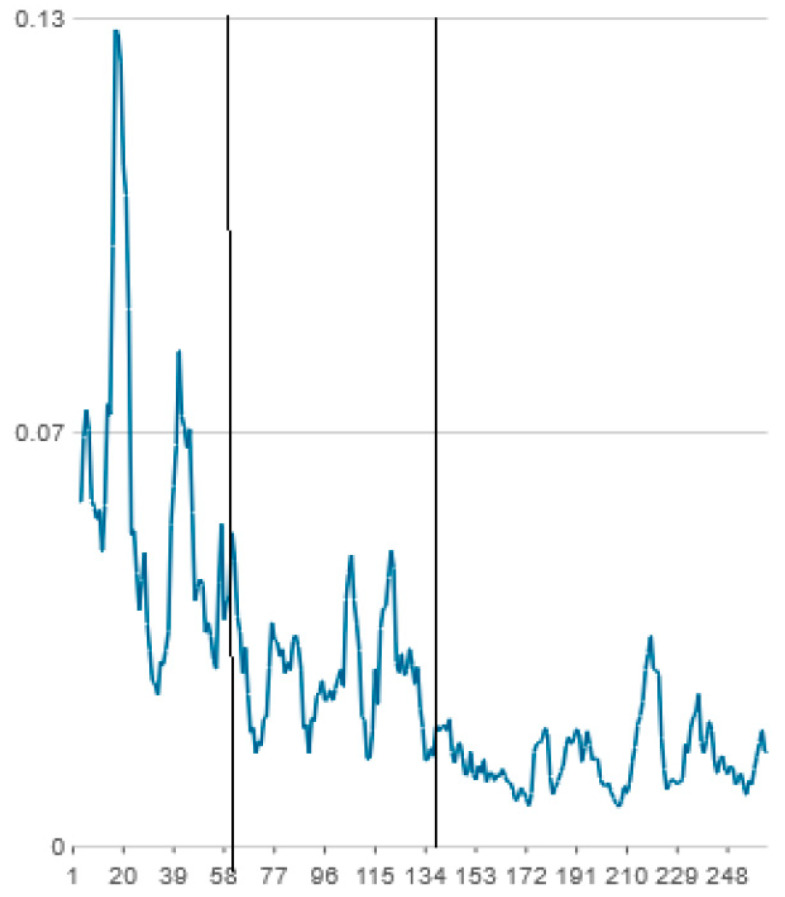
Dynamic complexity averaged over all items of the TPQ subset as used in this study. The right vertical line corresponds to the pattern transition as it was found in most of the time series (see also Figure 2), and the left vertical line corresponds to a less frequently found pattern transition occurring at the end of the MBSR program.

**Figure 6:**
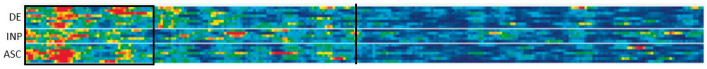
Complexity Resonance Diagram of the 20 items corresponding to the factors “Dysphoric Emotions” (DE), “insight/new perspectives” (INP), and “awareness/self-care” (ASC). The sequence of the items corresponds to Table 1. Blue colors: low complexity; red colors: high complexity. The highest dynamic complexity occurs during the first eight weeks of the monitoring period, corresponding to the period of the MBSR program (black frame). The vertical line corresponds to the pattern transition as it was found in most of the time series and the factor dynamics (see Figure 2).

As can be seen in the time series, the fluctuation intensity and complexity of the process are higher in the first part of the process, which corresponds to the MBSR program period. This impression is confirmed by the averaged dynamic complexity time series of the items (see Figure 5) and by the Complexity Resonance Diagram (see Figure 6). 

Beyond the overall reduction in complexity during the assessment period, it is evident that this is not a continuous process. The vertical lines in Figure 4 and Figure 5 indicate two steps of complexity reduction. The right vertical line in Figure 5 corresponds to the pattern transition as it was found in most of the time series (see also Figure 3); the left vertical line corresponds to a less frequently found pattern transition occurring at the end of the MBSR program.

Figure 6 shows the Complexity Resonance Diagram of the 20 items of the *Therapy Process Questionnaire* (TPQ) corresponding to the factors “Dysphoric Emotions” (IV), “insight/new perspectives” (V), and “awareness/self-care” (VII) (blue colors indicate low complexity, red colors indicate high complexity). The highest dynamic complexity occurs during the first eight weeks of the monitoring period, corresponding to the period of the MBSR program (black frame). The right vertical line of the frame corresponds to a pattern transition, as it was found in many but not all of the time series. The other pattern transition occurred about four months later, at about measurement point 135 in the middle of the assessment process. The recurrence plots of the factors and the item dynamics demonstrate the transitions, especially the stabilized dynamics from about measurement point 135 to the end of the monitoring period. This seems to be the dominant pattern transition.

**Figure 7:**
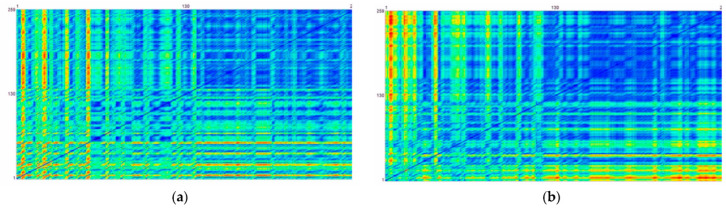
Colored recurrence plots of the change dynamics. (**a**) Item “Today, I felt anxious” (compare Figure 3a); (**b**) factor “awareness/self-care” (compare Figure 3c). The blue pixels at the right upper subarea of the recurrence plots indicate the stabilization of the process after the pattern transition, which could be seen in the factors and in most of the items.

Figure 7 shows the colored recurrence plots of the change dynamics of an item and a factor: (a) item “Today, I felt anxious” (compare Figure 3a); (b) factor “awareness/self-care” (compare Figure 2c). The blue pixels at the right upper subarea of the recurrence plots indicate the stabilization of the process after the pattern transition, which could be seen in the factors and in most of the items.

The pattern or phase transition of the time series was identified by the Pattern Transition Detection Algorithm. It revealed pattern transitions at about measurement point 135, during the six months of the assessment period and about four months after the end of the mindfulness program, and another, less accentuated pattern transition at the end of the program (Figure 3 and Figure 4). After the dominant transition, the complex, high-volatile dynamics changed to a more stable pattern with reduced anxiety and fewer feelings of tenseness and restlessness. Corresponding to this, mindfulness, happiness, lightheartedness, feeling comfortable in her body, and attentiveness to boundaries increased and stabilized (Figure 3), together with other items that are not illustrated by figures.

**Figure 8:**
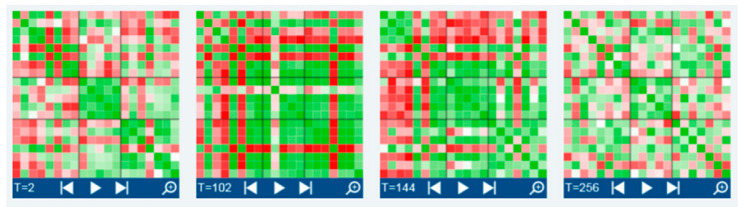
Four snapshots of correlation patterns (correlation matrices at time 2, 102, 144, and 256; window width: 7 measurement points). Each cell of the matrices depicts the correlation of a respective item with another item on a gradual green (positive correlation values, 0 < r <1) or red (negative correlation values, −1 < r < 0) scale (white cells correspond to a correlation of 0).

During the process, the patterns of the inter-item correlations changed through spontaneous jumps. In the *Synergetic Navigation System* (SNS), this is illustrated like a movie when dragging the correlation matrix (window width: 7 measurement points) over the time series. Figure 8 shows the snapshots of four correlation matrices at different time points (time 2, 102, 144, and 256; window width: 7 measurement points). Each cell of the matrices depicts the correlation of a respective item with another item on a gradual green (positive correlation values, 0 < r < 1) or red (negative correlation values, −1 < r < 0) scale (white cells correspond to a correlation of 0).

To obtain an impression of the feedback sessions, see Table 2 with some topics from the semi-structured interview.

In an *evaluation interview* 7 months after the end of the one-year assessment period (the protocol of the semi-structured interview is available upon request from M.E.H.), the participant confirmed the feasibility of continuous self-ratings by the *Therapy Process Questionnaire* (TPQ). The number of items in her TPQ was not too many, and the 10 min for accomplishing the self-rating and writing comments were experienced as an important time of self-reflection and becoming aware of her feelings, inner states, and actions. She appraised process monitoring as an important procedure for enhancing meta-awareness, successfully transferring mindfulness training to everyday life, and increasing her motivation for continuing her daily meditation exercises. The feedback sessions on the visualized processes in the SNS, together with her mindfulness teacher (T.M. and M.E.H.), were evaluated as very useful but should have been more frequent (e.g., once per week). During the feedback sessions, she learned more about her personal sense-making and the meaning of emotions. Looking at and discussing the graphs of the emotion items was helpful to accept instead of avoid the emotions and to become more aware of her feelings. Step by step, she developed an understanding of the interconnection of experiences that were represented by the items. She became aware that personal development takes time and cannot be accelerated and that worrying feelings or periods of stagnation are important and can be accepted to occur. Another important experience concerned the anticipation of answering the questions in the evening, just during the day. She reported anticipating how the assessment of herself in the questionnaire would look if she would accomplish or act in this or that way, which helped to manage her cognitions, emotions, and behavior during the day, to become more aware of herself, and to relate and respond to the present experience in a healthy way as much as possible.

Concerning the background of the “therapeutic alliance”, our participant felt understood from the beginning of the program. From the beginning of the daily assessments, she also felt that self-ratings make sense and daily questions are “more than statistics”. During the feedback sessions, she experienced that someone was really interested in her development.

In the retrospective interview, she could not concretely remember some specific triggers or life events associated with the pattern transition during the 6th month of the monitoring period. It seemed like a spontaneously emerging pattern transition. Interestingly, this was the sequence with the most frequent entries in the comment field during the whole monitoring period. She wrote short comments about mixed and fast-switching experiences and emotions, from grief and stress to joy and happiness, about different feelings (stress, lacking appreciation, but also proudness and joy at her workplace and with her children), and about receiving support from meditation exercises—a kind of ambiguity that is a well-known correlate of pattern transitions. To conclude, she would recommend combining MBSR programs with continuous high-frequency monitoring and feedback sessions on the visualized development pattern

## 4. Discussion

### 4.1. Dynamics of Mindfulness-Based Interventions as a Self-Organizing Process

The first aim of this case study was to contribute to the understanding of the process and mechanisms of change in mindfulness-based interventions (MBIs). The patterns of change during one year confirm the self-organization hypothesis of MBI dynamics. 

To the best of our knowledge, this case study is the first that describes changing patterns of multiple time series, which were produced by daily self-assessments during and after an MBSR program (assessment period: one year). This produced important and detailed information on the effects of mindfulness programs beyond pre-post comparisons. In the time series of most items, it became evident that the participant experienced progress in her well-being and also experienced less worrying and more positive emotions. She developed insight and new perspectives on her life, as well as more awareness of her body, needs, and limits. These results confirm findings on the positive effects of mindfulness programs in general and specifically for healthcare professionals [55], especially enhancing nurses’ coping with work-related stress [4]. Mindfulness as measured by the CHIME and self-compassion as measured by the SCS increased.

Far away from linear dose-dependent effects, we see spontaneous pattern transitions and changing complexity related to pattern transitions (comp. [37,41]). The figures illustrate the changing volatility and complexity of cognitive and emotional patterns (Figure 3, Figure 4, Figure 5, Figure 6 and Figure 7), changes in inter-item correlation patterns (Figure 8), and occurring phase transitions (Figure 3, Figure 4, Figure 5, Figure 6 and Figure 7). The most important result confirming the hypothesis on self-organization as a mechanism of mindfulness dynamics comes from the Pattern Transition Detection Algorithm. It detected one dominant and one secondary pattern or phase transition at specific times of the process. Discontinuous pattern transitions can be seen as the core feature of self-organizing dynamics in complex systems, independent of the “substrate” of the system [32]. In this case, we observed a stepwise decrease in the dynamic complexity of the system at both transition points (see Figure 5 and Figure 6), as well as a transition from more unstable and complex dynamics (transient dynamics in terms of the Recurrence Plot methodology) to more stable (recurrent in terms of RP) dynamics at the dominant transition point (Figure 7). Complexity, as captured by the dynamic complexity measure or by recurrence plots, represents the degree of instability of—in this case—the cognitions, emotions, and behavior of the concerned person. This may be due to a strong emotional involvement in the process of development, emotional insecurity or ambiguity, more or less successful coping with stress, or other reasons. As reported in the feedback sessions and in the final interview, she found a way out of stressful life circumstances to a more stable psychosocial functioning exactly at the time of the dominant pattern transition.

The main pattern transition occurred about four months after finishing the MBSR program. In terms of self-organization, it may be interesting to see that this dominant transition point was not related to or even “caused by” a specific input to the system, like a change in the setting, some interventions, or some life event. The participant continued mindfulness and meditation practice, which confirms existing knowledge of the effects of this practice and raises questions about the time structure and patterns of change in mindfulness-based interventions and contemplative practices. Grabovac [56] describes “stages of insight” as predictable developmental sequences of the Theravada Buddhist practice, with intense experiences and even psychiatric symptoms in some of the stages. Analayo [57] raises the “*… question of the extent to which progress to realization follows a ‘gradual‘ pattern, as against an unexpected ‘sudden’ breakthrough to awakening*” (p. 252). In his phenomenological study on the experiences of students during two-week and three-month intensive training periods, Kornfield [28] concludes: “*Meditation does not appear to be a linear learning or developmental process. Instead, the ‘mindfulness’ meditation appears to include periods of regression, restructuring and reintegration as part of the basic growth pattern*” (p. 53). Non-stationary effects challenge any assumptions about change occurring in a gradual, linear, and input-dependent way [39]. Instead, the importance of common factors (see the contextual model [58]) and the self-organization model of human change [25,59,60] are supported. 

### 4.2. Continuous Assessment and Systematic Feedback Support the Change Process

Our interview data confirm that systematic feedback supports the therapeutic process. This is not only reported by the participant but is also confirmed by the parallel continuity of daily self-assessments and mindfulness practice. “Feedback-informed treatment” or “feedback-driven therapy” realizes that data-based (process-related) interviews during the feedback sessions may have a beneficial impact on psychological treatments [61,62,63]. 

The participant reported on some psychological mechanisms contributing to the positive effects of her mindfulness practice in the feedback sessions and the evaluation session. Her own description was fitting to the IAA-model (intention, attention, attitude) of mindfulness [17]. From her own (first-person) perspective, the experienced intention contributes to mindfulness and provides knowledge on why to practice. Intentions relate to practice—to be mindful or compassionate or to be aware of what is going on [33], and by doing so, they modify mindful states. This has an impact on motivation and on the commitment to the MBSR program, to practice, and to engagement in the study. The daily process monitoring keeps up the focus of the mindfulness practice during the day. For the participant, it was helpful to anticipate the procedure of answering the questions just during the day. This anticipation contributed to a decentered perspective (decentering) and increased self-awareness and meta-awareness. Focusing on her experiences reduced self-judgements and ruminations. Decentered self-awareness influenced self-regulation in a positive way.

During the process, the working alliance [64,65] contributed to the process, its continuity, and the success of the SNS-based feedback. Feeling understood and supported—also in the feedback sessions—by the MBSR teacher strengthened the collaborative bond [63]. This was an important precondition for reaching the goals of stress reduction at her workplace and home and improving her self-love. 

The MBSR program provides further contextual factors for the development process. The atmosphere in the MBSR classroom is, in Winnicott’s terms, a “holding environment” [43] (compare the first generic principle of “stability conditions” [62]). Experiencing the mindfulness teacher embodying and modeling a compassionate attitude toward herself and others, together with loving-kindness meditations, increased her self-compassion. Self-inquiry [66] was facilitated by the monitoring process, which conveyed insight into the transient nature of subjective experiences. Self-inquiry and reflections on herself contributed to self-acceptance by exploring motives, needs, emotions, and thoughts. This was supported by the SNS-based feedback sessions.

In this context, we should distinguish between retrospective remembering of the day and present-moment awareness. Answering the process questions with a mindful and self-compassionate attitude may prevent the adverse effects of thinking about the day, which may trigger negative self-judgments and ruminations. A mindful state makes rumination less likely and reduces its impact [67]. Stress and negative states of self-compassion reduce awareness of mindfulness-related intentions [33]. When she lost her mindful intentions in stressful situations, she also lost self-awareness, self-regulation, and her capacity to take a decentered perspective.

### 4.3. Feasibility of High-Frequency Process-Monitoring and Process-Feedback

The results of this study replicate existing findings on the feasibility and compliance of high-frequency process monitoring over longer periods of time. Whereas these findings came from inpatient and outpatient psychotherapy [68], our participant was a healthy woman working in the health care system. During one year, beginning with an MBSR program and continuing with daily mindfulness practice, she filled in a subset of the items of the *Therapy Process Questionnaire* (TPQ) [44] at 262 days (71.8%) of the monitoring period. Also, she used the option to write diaries in the comment field of the questionnaire. She evaluated the monitoring procedure as helpful for the transfer of the mindfulness curriculum and mindfulness practice to everyday life. She recommended integrating monitoring procedures with mindful and compassionate support during feedback meetings into mindfulness programs and applying this to all users of mindfulness-based programs. The motivation of our participants was supported by the alliance and the bond with the MBSR teacher, but also by the feedback sessions.

The findings of the single case study correspond to the benefits of systematic monitoring and feedback, which are reported in psychotherapy (e.g., [69,70,71]). Opinion leaders denote it as “good practice” [72,73]. However, when discussing benefits or burdens, we should be aware that there are different ways of realizing process monitoring in real-world settings. There are several considerations in this context. One concerns the sampling rate (e.g., assessment at the sessions or assessments every day; what is high-frequency equidistant time sampling?). Another consideration is the frequency and aim of feedback sessions (e.g., only for reacting to potential deteriorations or at-risk developments suggesting clinical support tools or—different from this—continuously reflecting on change processes). The third concerns the philosophy of change dynamics (expecting “on track” or “not on track” and individualized dynamics) and, in consequence, how the procedure is explained. Finally, there is a question about whether standardized or personalized questionnaires are used in the process. Of course, therapists have to be motivated and convinced to realize monitoring and feedback. They have to be skilled and trained (at our institute, a training program in therapy feedback is offered once per year). Depending on these ways to realize it and on the preconditions, process monitoring may be burdensome in some cases, but the benefits seem to outperform the burdens in many ways. Clients experience it as motivating for change, as helpful for self-reflection, as supporting the client–therapist alliance, as catalyzing the change process, or as contributing to learning emotion regulation and self-management (e.g., [69,74], also owning still unpublished data from a recent user survey). Referring to many years of feedback practice in different settings, clients experience self-assessments and writing diaries as interventions. 

With and without electronic or app-based feedback, a relationship concept between the client and therapist is important where they agree on a narrative that is recognizable for both and offers a starting point for treatment [58]. To arrive at this point of agreement, information on a client’s narrative is gathered by having verbal conversations, designing case formulations in a co-creative process, and, based on this, working with feedback tools and feedback interviewing in the process [62]. 

In consequence, the way electronic diary data are collected (pre-)processed and the statistical models selected for analyses largely influence the treatment target based on feedback provided (e.g., [75,76]). Equidistant, high-frequency monitoring, as it is realized in this study, allows for nonlinear time series analysis, providing insight into the features of self-organization and chaotic dynamics, which is different from linear assumptions as therapies should move on track (for a comparison, see [36]) or should follow auto-regression statistics. So, perhaps more important than monitoring or not is the scientific paradigm, which is used for understanding change.

### 4.4. Limitations

Here, we report on a single case study whose results have to be replicated by further research. Results from single case studies should not be generalized. To further confirm the hypothesis of self-organized change processes, patterns of change should be observed in larger clinical and non-clinical samples. Also, the feasibility of app-based self-monitoring cannot be proven by a *n* = 1 study, but the results are in line with other findings from inpatient and outpatient psychotherapy [68].

The applied assessment instruments on mindfulness and self-compassion were not developed to mirror dynamics. The application of the CHIME only produced one-point measures at the beginning and at the end of the MBSR training and the end of the monitoring program about 10 months later. The application of the SCS produced pre and post-results, as well as monthly states. Other than this, the TPQ allowed daily measures, and given this high-frequency sampling rate, it produced fine-grained multiple-time series that can be analyzed by different nonlinear methods. Results from these methods can be interpreted within the framework of self-organization theory. Out of the field of MPs, different studies gave evidence of nonlinear, chaotic, and self-organizing dynamics in clinical change (e.g., [25,37]), which supports the assumption that self-organization is a more general mechanism, not restricted to mindfulness dynamics. 

Another limitation concerns the measurement of mindfulness by self-reports [15]. Self-reports depend on the interpretation of the item scales, and the results depend on the subjective interpretation and understanding. One more point is that within-subject dynamics were not compared with interpersonal differences or population dynamics. This may be a limitation, but it is also a consequence of the lack of ergodicity in most psychological system dynamics. More than this, we can assume changing frames of perception and interpretation of psychological experiences over time [77]. 

Finally, we cannot exclude that the changes in complexity we observed during the monitoring period are due to an initial elevation bias resulting from repeated self-assessments [78]. This would be a measurement bias not mirroring any mindfulness-related dynamics, exercise effects, or features of self-organization. To exclude an initial elevation bias, a period before the mindfulness-based treatment should have been assessed. On the other side, we found discontinuous changes in complexity that were not predicted by the bias of merely repeated self-assessments. One occurred after the mindfulness group training, and another one occurred four months later. If complexity reduction were a linear time effect, this should not occur. Usually, self-organization is not a simple reaction to therapeutic interventions, nor is it a mere time effect, but it may result from intra-systemic dynamics reaching certain thresholds. In many cases, complexity is not only decreased but also increased (e.g., [68]). 

### 4.5. Perspectives

Combining mindfulness-based interventions (MBIs) with app-based process monitoring and feedback interviewing based on shared attention to the diagrams provided by the SNS requires a computer screen at the workplace of the therapist, coach, or mindfulness teacher. This can be easily realized in rehabilitation clinics, which integrate MBIs with group and individual psychotherapy, or in outpatient psychotherapy or coaching settings. SNS-based process monitoring may be part of the internet- or app-based online formats of MBIs. The feasibility and effectiveness of feedback-driven MBIs could even be greater than in face-to-face settings alone. Following experiences in many settings, the visualization of the client’s own processes is motivating and catalyzes the process of development. 

Continuous shared process monitoring allows for the individualization of programs and process sensitivity concerning timing, intensity, and quality of interventions. In a program of mindfulness-integrated cognitive behavior therapy, Cayoun [79] proposed to proceed to the next step of the program only when some conditions and steps were realized. Using process monitoring in research, we obtain the option to see mindfulness as a psychological process in varying states. Following Bishop et al. [16], mindfulness is much closer to state dynamics than a trait. Blanke and Brose [80] developed a questionnaire to assess the multidimensionality of mindfulness at both the trait and the state level *(Multidimensional State Mindfulness Questionnaire; MSMQ)*. Another process-oriented mindfulness questionnaire is the *Mindfulness Process Questionnaire (MPQ)* by Erisman and Roemer [27].

To find the “middle way” between “not enough” and “too much of a good thing”, Britton [81] proposed a non-monotonic framework that should help to maximize effectiveness and minimize the harms of MBIs. To explain the non-monotonic effects of mindfulness-related practice, she proposed an inverted U-shaped relationship between mindfulness-related processes and well-being. Mediating variables like baseline characteristics can influence the effects of practice. Here comes systematic, continuous monitoring of the stage in order to detect individual adverse effects just in time. Reynolds et al. [82] reported increased symptoms of distress after a brief mindfulness intervention in an RCT with chemotherapy patients. They conclude that: “*… these results suggest that regular monitoring of psychological status during mindfulness training is warranted—not simply pre- and post-intervention as is common*”.

Psychotherapy “*… can be seen as a dynamic and adaptive attempt to provide conditions for self-organized pattern transitions in the biopsychosocial system of participants or clients*” (p. 18) [62]. For mindfulness-based interventions, Santorelli [43] states in a similar way: “*… we are trusting in the nobility and inborn genius of human beings and creating a learning environment in which this nobility and genius might emerge and be known by them*” (p. 52). Process monitoring supports exploring barriers and optimizes learning environments.

## Figures and Tables

**Table 1:** Items for daily self-ratings.

Dysphoric Emotions (DE)
Today, I felt joy (r)
Today, I experienced moments of happiness and lightheartedness (r)
Today, my self-esteem was … (r)
Today, I felt anxious
Today, I felt sad
Today, I felt guilty
Today, I felt angry
Today, I felt shame
**Insight/new perspectives (INP**)
Today, I became aware of relations that were not clear to me before
Today, I worked on things that were new and unusual for me
Today, I had new insights about how to better deal with my life circumstances
Today, I gained insight into how my thoughts, feelings, and behavior influence each other
I now understand myself and my problems better
**Awareness/self-care (ASC)**
Today, I was aware of my own needs
Today, I paid attention to my bodily signals
Today, I paid attention to my boundaries/limits
Today, I treated myself with care and mindfully
Today, I felt tense and restless (r)
Today, I felt comfortable in my body
Today, I can cope with my emotions

Note: The items for daily self-ratings are a subset of the Therapy Process Questionnaire (TPQ) adapted for non-clinical settings and were approved by our participant as useful and sense-making. The clusters are similar to but not identical to the three factors of the TPQ. Some of the items have to be reversed for calculating the factor values (r). The answer scales are visual analog scales, e.g., from “not at all” to “very strong”.

**Table 2:** Topics and exemplified answers of the feedback interviews.

Topics	Exemplified Answers
Actual mood, important mental and situation-related inner and external *experiences* since the last feedback session	Feeling less supported after the end of the MBSR program; at work, less anxious, especially when it was easier for her to ask for and accept help; she developed a more positive attitude towards her body with more pleasant sensations
Reaching *goals*	More self-love and self-compassion; difficulties with connectedness; more awareness of ways to cope with difficult situations in daily life
Experiences with mindfulness *practice*	Difficulties with the body scan—feelings of freezing; forgetting informal practice during work; feeling more alive with adapted sentences for the loving-kindness meditation
*Transfer and integration* of mindfulness in daily life (work and family)	Remembers recent situations at work when she is mindful, adding new situations while being aware of recognizing her own strengths and skills
*Barriers*	Difficulties with mind-wandering during the exercises; she felt “not good enough“; acknowledging herself for successes too much could diminish further efforts
Experiences with *daily assessments*	In difficult situations during the day, she thinks about how she will fill out the assessment in the evening; this helps her remember some intentions. She has difficulties answering questions (e.g., how exactly does anger feel?); sometimes unwilling to answer the questions when she experiences two meanings in one question (e.g., tense *and* restless)
Working on *insights* given by experiences	What are the connections between needs and feelings? What are her needs? Potential functions of anger
Looking together at the *time series diagrams* of interest (e.g., anxiety, joy, guilt, shame, anger, awareness of needs)	She tries to understand the peaks of the graphs, elations, and senses of delight (e.g., during periods of leisure time or feeling joy during a skiing excursion with her daughter), downs (e.g., around menstruation or during caregiving for a relative), and periods of fluctuations in the diagrams; for a longer period, she felt more stable at a new level of personal integrity
Changing *goals*	She felt an attitude of acceptance towards feelings; seeing her own contribution to successes; finding a balance between polarities (e.g., movement and rest; self and others)
Changing *practice*	Movement of fingers in the body scan when feelings of freezing arise; adding sentences to the loving-kindness meditation about safety; remembering a sense of warmth and loveliness around the heart; setting anchors for informal practice

## Data Availability

The data presented in this study are available upon request (due to protection of privacy) from the corresponding author.

## References

[1] Goldberg S.B., Tucker R.P., Greene P.A., Davidson R.J., Wampold B.E., Kearney D.J., Simpson T.L. (2018). Mindfulness-based interventions for psychiatric disorders: A systematic review and meta-analysis. Clin. Psychol. Rev..

[2] Wielgosz J., Goldberg S.B., Kral T.R., Dunne J.D., Davidson R.J. (2019). Mindfulness meditation and psychopathology. Ann. Rev. Clin. Psychol..

[3] Baer R.A., Carmody J., Hunsinger M. (2012). Weekly change in mindfulness and perceived stress in a mindfulness-based stress reduction program. J. Clin. Psychol..

[4] Smith S.A. (2014). Mindfulness-based stress reduction: An intervention to enhance the effectiveness of nurses’ coping with work-related stress. Int. J. Nurs. Knowl..

[5] Schuman-Olivier Z., Trombka M., Lovas D.A., Brewer J.A., Vago D.R., Gawande R., Dunne J.P., Lazar S.W., Loucks E.B., Fulwiler C. (2020). Mindfulness and behavior change. Harv. Rev. Psychiatry.

[6] Butler L.D., Mercer K.A., McClain-Meeder K., Horne D.M., Dudley M. (2019). Six domains of self-care: Attending to the whole person. J. Hum. Behav. Soc. Environ..

[7] Christopher J.C., Maris J.A. (2010). Integrating mindfulness as self-care into counselling and psychotherapy training. Couns. Psychother. Res..

[8] Coaston S.C. (2017). Self-care through self-compassion: A balm for burnout. Prof. Couns..

[9] Brown K.W., Ryan R.M. (2003). The benefits of being present: Mindfulness and its role in psychological well-being. J. Pers. Soc. Psychol..

[10] Schultz P.P., Ryan R.M., Ostafin B.D., Robinson M.D., Meier B.P. (2015). The “why”, “what”, and “how” of healthy self-regulation: Mindfulness and well-being from a self-determination theory perspective. Handbook of Mindfulness and Self-Regulation.

[11] Fjorback L.O., Arendt M., Ornbøl E., Fink P., Walach H. (2011). Mindfulness-based stress reduction and mindfulness-based cognitive therapy: A systematic review of randomized controlled trials. Acta Psychiatr. Scand..

[12] Kabat-Zinn J. (1982). An outpatient program in behavioral medicine for chronic pain patients based on the practice of mindfulness meditation: Theoretical considerations and preliminary results. Gen. Hosp. Psychiatry.

[13] Segal Z.V., Williams J.M., Teasdale J. (2002). Mindfulness-Based Cognitive Therapy for Depression: A New Approach to Preventing Relapse.

[14] Kabat-Zinn J. (2011). Some reflections on the origins of MBSR, skillful means, and the trouble with maps. Contemp. Buddhism.

[15] Grossman P. (2019). On the porosity of subject and object in “mindfulness” scientific study: Challenges to “scientific” construction, operationalization and measurement of mindfulness. Curr. Opin. Psychol..

[16] Bishop S.R., Lau M., Shapiro S., Carlson L., Anderson N.D., Carmody J., Segal Z.V., Abbey S., Speca M., Velting D. (2004). Mindfulness: A proposed operational definition. Clin. Psychol. Sci. Pract..

[17] Shapiro S.L., Carlson L.E., Astin J.A., Freedman B. (2006). Mechanisms of mindfulness. J. Clin. Psychol..

[18] Safran J.D., Segal Z.V. (1990). Interpersonal process in cognitive therapy.

[19] Bernstein A., Hadash Y., Lichtash Y., Tanay G., Shepherd K., Fresco D.M. (2015). Decentering and related constructs. A critical review and metacognitive processes model. Perspect. Psychol. Sci..

[20] Bernstein A., Hadash Y., Fresco D.M. (2019). Metacognitive processes model of decentering: Emerging methods and insights. Curr. Opin. Psychol..

[21] Bergomi C., Tschacher W., Kupper Z. (2013). Measuring mindfulness: First steps towards the development of a comprehensive mindfulness scale. Mindfulness.

[22] Shen C.Y., Midgley G. (2007). Toward a buddhist systems methodology 1: Comparisons between buddhism and systems theory. Syst. Pract. Action Res..

[23] Macy J. (1991). Mutual Causality in Buddhism and General Systems Theory.

[24] Bertalanffy L. (1950). The theory of open systems in physics and biology. Science.

[25] Haken H., Schiepek G. (2010). Synergetik in der Psychologie: Selbstorganisation verstehen und gestalten, 2. Aufl. Synergetics in Psychology: Understanding and Supporting Self-Organization.

[26] Tschacher W., Lienhard N. (2021). Mindfulness is linked with affectivity in daily life: An experience-sampling study with meditators. Mindfulness.

[27] Erisman S.M., Roemer L. (2012). A preliminary investigation of the process of mindfulness. Mindfulness.

[28] Kornfield J. (1979). Intensive insight meditation: A phenomenological study. J. Transpers. Psychol..

[29] Varela F.J., Thompson E., Rosch E. (1991). The Embodied Mind: Cognitive Science and Human Experience.

[30] Rosch E., Varela F.J., Thompson E., Rosch E. (2016). Introduction to the revised Edition. The Embodied Mind: Cognitive Science and Human Experience, revised ed..

[31] Thompson E., Varela F.J., Thompson E., Rosch E. (2016). Introduction to the revised Edition. The Embodied Mind: Cognitive Science and Human Experience, revised ed..

[32] Haken H. (2004). Synergetics. Introduction and Advanced Topics.

[33] Suelmann H., Brouwers A., Snippe E. (2018). Explaining variations in mindfulness levels in daily life. Mindfulness.

[34] Andreotti E., Congard A., Le Vigouroux S., Dauvier B., Illy J., Poinsot R., Antoine P. (2018). Rumination and mindlessness processes: Trajectories of change in a 42-day mindfulness-based intervention. J. Cogn. Psychother..

[35] Aizik-Reebs A., Shoham A., Hadash Y., Bernstein A. (2021). A network modeling approach to mindfulness mechanisms: A proof-of-concept investigation. Mindfulness.

[36] Schiepek G., Gelo O., Viol K., Kratzer L., Orsucci F., de Felice G., Stöger-Schmidinger B., Sammet I., Aichhorn W., Schöller H. (2020). Complex individual pathways or standard tracks? A data-based discussion on the trajectories of change in psychotherapy. Couns. Psychother. Res..

[37] Olthof M., Hasselman F., Strunk G., van Rooij M., Aas B., Helmich M.A., Schiepek G., Lichtwarck-Aschoff A. (2019). Critical fluctuations as an early-warning signal for sudden gains and losses in patients receiving psychotherapy for mood disorders. Clin. Psychol. Sci..

[38] Molenaar P.C.M. (2013). On the necessity to use person-specific data analysis approaches in psychology. Europ. J. Develop. Psychol..

[39] Hayes A.M., Laurenceau J.-P., Feldman G., Strauss J.L., Cardaciotto L. (2007). Change is not always linear: The study of nonlinear and discontinuous patterns of change in psychotherapy. Clin. Psychol. Rev..

[40] Westerman M.A. (2009). What can we learn from case studies? More than most psychologists realize. Pragmat. Case Stud. Psychother..

[41] Viol K., Schöller H., Kaiser A., Fartacek C., Aichhorn W., Schiepek G. (2022). Detecting pattern transitions in psychological time series—A validation study on the pattern transition detection algorithm (PTDA). PLoS ONE.

[42] Kabat-Zinn J. (1990). Full catastrophe living. Using the Wisdom of Your Body and Mind to Face Stress, Pain, and Illness.

[43] Santorelli S.F., McCown D., Reibel D., Micozzi M.S. (2016). Remembrance: Dialogue and inquiry in the MBSR classroom. Resources for Teaching Mindfulness.

[44] Schiepek G., Stöger-Schmidinger B., Kronberger H., Aichhorn W., Kratzer L., Heinz P., Viol K., Lichtwarck-Aschoff A., Schöller H. (2019). The therapy process questionnaire. Factor analysis and psychometric properties of a multidimensional self-rating scale for high-frequency monitoring of psychotherapeutic processes. Clin. Psychol. Psychother..

[45] Raes F., Pommier E., Neff K.D., van Gucht D. (2011). Construction and factorial validation of a short form of the self-compassion scale. Clin. Psychol. Psychother..

[46] Hupfeld J., Ruffieux N. (2011). Validierung einer deutschen Version der Self-Compassion Scale (SCS-D). Z. Klin. Psychol. Psychother..

[47] Coroiu A., Kwakkenbos L., Moran C., Thombs B., Albani C., Bourkas S., Zenger M., Brahler E., Körner A. (2018). Structural validation of the self-compassion scale with a german general population sample. PLoS ONE.

[48] Schiepek G., Strunk G. (2010). The identification of critical fluctuations and phase transitions in short term and coarse-grained time series—A method for the real-time monitoring of human change processes. Biol. Cybern..

[49] Marwan N., Romano M.C., Thiel M., Kurths J. (2007). Recurrence plots for the analysis of complex systems. Phys. Rep..

[50] Webber C.L., Marwan N. (2015). Recurrence Quantification Analysis: Theory and Best Practices.

[51] Schiepek G., Schöller H., de Felice G., Steffensen S.V., Skaalum Bloch M., Fartacek C., Aichhorn W., Viol K. (2020). Convergent validation of methods for the identification of phase transitions in time series of empirical and model systems. Front. Psychol..

[52] Killick R., Fearnhead P., Eckley I.A. (2012). Optimal detection of change points with a linear computational cost. J. Am. Stat. Assoc..

[53] Cohen L. (1989). Time-frequency distributions. A review. Proc. IEEE.

[54] Sundar A. (2023). Time Frequency Distribution of a Signal Using S-Transform (Stockwell Transform). MATLAB Central File Exchange. https://www.mathworks.com/matlabcentral/fileexchange/51808-time-frequency-distribution-of-a-signal-using-s-transform-stockwell-transform.

[55] Kriakous S.A., Elliott K.A., Lamers C., Owen R. (2021). The effectiveness of mindfulness-based stress reduction on the psychological functioning of healthcare professionals: A systematic review. Mindfulness.

[56] Grabovac A. (2015). The stages of insight: Clinical relevance for mindfulness-based interventions. Mindfulness.

[57] Analayo (2003). Satipatthana. The Direct Path to Realization.

[58] Wampold B.E., Imel Z.E. (2015). The Great Psychotherapy Debate: The Evidence for What Makes Psychotherapy Work, 2nd ed.

[59] Schiepek G., Viol K., Aichhorn W., Hütt M.T., Sungler K., Pincus D., Schöller H. (2017). Psychotherapy is chaotic—(not only) in a computational world. Front. Psychol..

[60] Schöller H., Viol K., Aichhorn W., Hütt M.T., Schiepek G. (2018). Personality development in psychotherapy: A synergetic model of state-trait dynamics. Cogn. Neurodyn..

[61] Bovendeerd B., de Jong K., de Groot E., Moerbeek M., de Keijser J. (2022). Enhancing the effect of psychotherapy through systematic client feedback in outpatient mental healthcare: A cluster randomized trial. Psychother. Res..

[62] Schiepek G., Eckert H., Aas B., Wallot S., Wallot A. (2015). Integrative Psychotherapy: A Feedback-Driven Dynamic Systems Approach.

[63] Brattland H., Koksvik J.M., Burkeland O., Klöckner C.A., Lara-Cabrera M.L., Miller S.D., Wampold B., Ryum T., Iversen V.C. (2019). Does the working alliance mediate the effect of routine outcome monitoring (ROM) and alliance feedback on psychotherapy outcomes? A secondary analysis from a randomized clinical trial. J. Couns. Psychol..

[64] Bordin E.S. (1979). The generalizability of the psychoanalytic concept of the working alliance. Psychother. Theor. Res. Pract..

[65] Wampold B.E. (2015). How important are the common factors in psychotherapy? An update. World Psychiatry.

[66] Dahl C.J., Lutz A., Davidson R.J. (2015). Reconstructing and deconstructing the self: Cognitive mechanisms in meditation practice. Trends Cogn. Sci..

[67] Blanke E.S., Schmidt M.J., Riediger M., Brose A. (2020). Thinking mindfully: How mindfulness relates to rumination and reflection in daily life. Emotion.

[68] Michaelis R., Edelhäuser F., Hülsner Y., Trinka E., Viol K., Schiepek G. (2021). High frequency monitoring of personalized psychological variables during outpatient psychotherapy in people with seizures: An uncontrolled feasibility study. Epilepsy Behav..

[69] de Jong K., Conijn J.M., Gallagher R.A.V., Reshetnikova A.S., Heij M., Lutz M.C. (2021). Using progress feedback to improve outcomes and reduce drop-out, treatment duration, and deterioration: A multilevel meta-analysis. Clin. Psychol. Rev..

[70] Delgadillo J., Overend K., Lucock M., Groom M., Kirby N., McMillan D., Gilbody S., Lutz W., Rubel J.A., de Jong K. (2017). Improving the efficiency of psychological treatment using outcome feedback technology. Behav. Res. Ther..

[71] Shimokawa K., Lambert M.J., Smart D.W. (2010). Enhancing treatment outcome of patients at risk of treatment failure: Meta-analytic and mega-analytic review of a psychotherapy quality assurance system. J. Consult. Clin. Psychol..

[72] Lambert M.J. (2017). Maximizing psychotherapy outcome beyond evidence-based medicine. Psychother. Psychosom..

[73] Wampold B. (2015). Routine outcome monitoring: Coming of age with the usual developmental challenges. Psychotherapy.

[74] McClintock A.S., Perlman M.R., McCarrick S.M., Anderson T., Himawan L. (2017). Enhancing psychotherapy process with common factors feedback: A randomized, clinical trial. J. Couns. Psychol..

[75] Bastiaansen J.A., Kunkels Y.K., Blaauw F.J., Boker S.M., Ceulemans E., Chen M., Chow S.-M., de Jonge P., Emerencia A.C., Epskamp S. (2020). Time to get personal? The impact of researchers choices on the selection of treatment targets using the experience sampling methodology. J. Psychosom. Res..

[76] Gual-Montolino P., Martinez-Borba V., Bretón-López J.M., Osma J., Suso-Ribera C. (2020). How are information and communication technologies supporting routine outcome monitoring and measurement-based care in psychotherapy? A systematic review. Int. J. Environ. Res. Public Health.

[77] Molenaar P.C.M., Campbell C.G. (2009). The new person-specific paradigm in psychology. Curr. Dir. Psychol. Sci..

[78] Shrout P.E., Stadler G., Lane S.P., McClure M.J., Jackson G.L., Clavél F.D., Iida M., Gleason M.E.J., Xu J.H., Bolger N. (2017). Initial elevation bias in subjective reports. PNAS Plus.

[79] Cayoun B.A. (2018). The Clinical Handbook of Mindfulness-Integrated Cognitive Behavior Therapy: A Step-by-Step Guide for Therapists.

[80] Blanke E.S., Brose A. (2017). Mindfulness in daily life: A multidimensional approach. Mindfulness.

[81] Britton W.B. (2019). Can mindfulness be too much of a good thing? The value of a middle way. Curr. Opin. Psychol..

[82] Reynolds L.M., Bissett I.P., Porter D., Consedine N.S. (2017). A brief mindfulness intervention is associated with negative outcomes in a randomized controlled trial among chemotherapy patients. Mindfulness.

